# Meta-analysis of studies using statins as a reducer for primary liver cancer risk

**DOI:** 10.1038/srep26256

**Published:** 2016-05-20

**Authors:** Guo-Chao Zhong, Yan Liu, Yuan-Yuan Ye, Fa-Bao Hao, Kang Wang, Jian-Ping Gong

**Affiliations:** 1 Department of Hepatobiliary Surgery, The Second Affiliated Hospital of Chongqing Medical University, Chongqing 400010, China; 2 Department of Gastroenterology, The Fifth People’s Hospital of Chengdu, Chengdu 611130, China; 3 Department of Laboratory Medicine, The Second Affiliated Hospital of Chongqing Medical University, Chongqing 400010, China; 4 Department of Pediatric Surgery, Children’s Hospital of Chongqing Medical University, Chongqing 400014, China; 5 Department of Breast and Endocrine Surgery, The First Affiliated Hospital of Chongqing Medical University, Chongqing 400016, China

## Abstract

A protective effect of statins on primary liver cancer (PLC) risk has been suggested. However, issues about the dose–response relationship, the protective effect of individual statins, and PLC risk reduction among at-risk populations remain unsolved. Therefore, a meta-analysis was conducted. PubMed and EMBASE were searched for studies providing the risk ratio (RR) on statins and PLC risk. Summary RRs were calculated using a random-effects model. Twenty-five studies were identified. Stain use was significantly associated with a reduced risk of PLC (RR = 0.60, 95% confidence interval (CI) = 0.53**–**0.69). The summary RR for every additional 50 cumulative defined daily doses per year was 0.87 (95% CI = 0.83**–**0.91). Evidence of a non-linear dose–response relationship between statins and PLC risk was found (*P*_non-linearity_ < 0.01). All individual statins significantly reduced PLC risk, and the risk reduction was more evident with rosuvastatin. The inverse association between statins and PLC risk remained among populations with common risk factors. Subgroup analyses revealed more significant reduction in PLC risk by statins in high- versus non-high-risk populations (*P*_interaction_ = 0.02). Overall, these findings add to our understanding of the association between statins and PLC risk. Whether statin use is causally associated with a reduced risk of PLC should be further studied.

Primary liver cancer (PLC) is the sixth most frequently diagnosed cancer worldwide^1^, representing approximately 6% of all new cancer cases^2^. Epidemiological studies show that the five-year relative survival rate of PLC in the USA was approximately 18%^3^, and the age standardized 5-year relative survival in China was approximately 10%^4^. These data reveal the poor prognosis of PLC, and thus PLC becomes the second leading cause of cancer-related death^1^. Statins are among the most widely prescribed medications globally because of their well-established efficacy and safety in the prevention and management of cardiovascular diseases^5^. In addition to the cardiovascular benefits, mounting evidence has suggested the chemopreventive potential of statins in the oncological field^6^.

Recently, statin use has been found to be associated with decreased risks of some cancers, including colorectal cancer^7^, ovarian cancer^8^, esophageal cancer^9^, gastric cancer^10^ and PLC^11^. However, as for statin use and PLC risk, there are several unsolved issues. First, whether there is a dose–response relationship between statin use and PLC risk is unclear. Some studies found that statin use was associated with a reduced risk of PLC in a dose–dependent manner^12^,^13^,^14^,^15^,^16^, whereas others failed to find a dose–response relationship between statin use and PLC risk^17^,^18^,^19^. Moreover, the shape of the dose–response relationship on this topic has not been described. Second, inverse^19^,^20^ and null^15^,^18^,^21^,^22^,^23^ associations with PLC risk have been reported for pravastatin, a commonly prescribed statin in clinical practice. This fact raises an issue of whether individual statin use is still associated with a reduced risk of PLC. Furthermore, which statin has the most pronounced protective effect on PLC risk is largely unknown. Third, previous meta-analyses^11^,^24^,^25^ did not investigate the association between statin use and PLC risk in subjects with common risk factors, including diabetes, hepatitis B virus (HBV) infection and hepatitis C virus (HCV) infection. It is possible that at-risk populations enjoy more benefits from statin chemoprevention than general populations^26^,^27^. In addition to these unsolved issues, previous meta-analyses^11^,^24^,^25^ are limited by the inclusion of limited studies, resulting in incompleteness and low power of their subgroup analyses. In fact, a recent meta-analysis (involving a total of 12 studies)^11^ included around half of studies currently available. In light of these considerations, we conducted an overall and dose–response meta-analysis of published studies to clarify the aforementioned unsolved issues and address limitations in previous studies.

## Methods

### Search strategy and study selection

We performed this meta-analysis and reported its results according to the statement of the Meta-analysis Of Observational Studies in Epidemiology^28^. A comprehensive electronic search of PubMed and EMBASE databases was conducted from their inception to March 2016, without any restrictions. The search strategy is shown in detail in the Supplementary List S1. The reference lists of included studies and relevant reviews were checked manually for identifying additional citations. If necessary, the original authors were contacted to obtain extra information through e-mails.

Studies were considered for inclusion if they were observational studies (cohort, nested case-control or case-control studies) or post hoc analyses of randomized controlled trials (RCTs) where risk estimate on the association of statin use with PLC risk was available. Two investigators (K.W. and F.B.H.) employed a two-stage method to conduct study screening independently. At the first stage, titles and abstracts were scrutinized for excluding obviously ineligible studies. At the second stage, the full text was read carefully for further excluding ineligible studies. Any disagreements on the eligibility of studies were resolved by discussion.

### Data extraction and quality assessment

Data extraction was performed by one investigator (K.W.), and was then checked independently for accuracy by another investigator (F.B.H.). The following information was extracted: first author, publication year, study location, study period, study design, study population, participants’ age, sample size, data source, definition of non-user, follow-up duration and statin dose where available, maximally adjusted risk estimate with corresponding 95% confidence interval (CI), and adjustment factors. Crude risk estimate with 95% CI was extracted when adjusted risk estimate was not available.

Two investigators (K.W. and F.B.H.) assessed the quality of included studies independently using the Newcastle-Ottawa quality assessment scale. Each study could be awarded to a maximum of 9 stars after evaluating its 3 aspects (selection, comparability, and outcome). A study with 7 or more stars was considered to be of high quality. Post hoc analysis of RCTs was treated as cohort studies to achieve quality assessment. Any disagreements on the results of data extraction and quality assessment were resolved by discussion.

### Statistical analysis

A random-effects model was used to pool the risk estimate from each study. Risk ratio (RR) was employed as a common measure of the association between statin use and PLC risk. Both hazard ratio and odds ratio were directly regarded as equivalent to RR. When only risk estimates stratified by type of statins^21^ or sex^29^ were available, we pooled these stratum data to yield an average risk estimate using a random-effects model. The Cochrane’s Q statistic (*P*_heterogeneity_ < 0.10 suggesting statistically significance) and the *I*^*2*^ statistic (*I*^*2*^ > 75.0% representing substantial heterogeneity, 50.0% ≤ *I*^*2*^ ≤ 75.0% representing moderate heterogeneity, *I*^*2*^ < 50% representing low heterogeneity) were adopted to qualitatively and quantitatively evaluate heterogeneity across studies, respectively.

On the basis of specific statin dose, distribution of cases and person years or controls, and adjusted RRs with 95% CIs, a two-stage dose–response meta-analysis was conducted to determine whether higher statin dose was associated with a lower risk of PLC. First, we used the generalized least square regression proposed by Orsini *et al*.^30^ to obtain study-specific slopes (linear trends) and 95% CIs for every 50 cumulative defined daily doses (cDDDs) per year increment in statin dose within each study from the natural logs of adjusted RRs and CIs across categories of statin dose. Then, we pooled them using a random-effects model to obtain an overall risk estimate. The defined daily dose (DDD) is a dose unit for statins, and refers to “the assumed average maintenance dose per day for a drug used for its main indication in adults”; the cDDD refers to the sum of dispensed DDDs of any statins during exposure period. Since all included studies provided statin dose as range, we designated the midpoint of each range as the assigned dose. If the highest range was open-ended, we assumed that it shared the same width as the preceding range. If the lowest range was open-ended, we assumed that the lower limit was equal to zero. For one study^12^ whose authors provided a cumulative dose of statins, we transformed it into cDDD under assumptions that the average DDD for statins was 25 mg, and the duration of statin use was the length of study period. To examine the robustness of the results from the two-stage dose–response meta-analysis, we performed a random-effects meta-analysis comparing the highest versus the lowest category of statin dose, which additionally included studies being not eligible for the two-stage dose–response meta-analysis. Restricted cubic spline function with 4 knots at the 5th, 35th, 65th and 95th percentiles was employed to identify any potential non-linear dose–response association between statin use and PLC risk. A *P*_non-linearity_ was obtained by testing the null hypothesis that the regression coefficients of both the second and the third spline are equal to zero^31^.

To check the stability of the pooled results and to identify the possible sources of heterogeneity, diverse sensitivity analyses were conducted using the following methods: ignoring a single study in turn, repeating meta-analysis through a fixed-effects model and using unadjusted risk estimates, and applying various eligibility criteria. To identify potential effect modifiers, prespecified subgroup analyses were conducted stratified by study design, source of subjects, sex, individual age, sample size, study location, risk level of developing PLC, study quality, adjustment for diabetes, HCV, or HBV, and level of adjustment for confounders. A *P*_interaction_ for difference between subgroups was calculated using meta-regression. Sensitivity and subgroup analyses were carried out based on the overall meta-analysis concerning statin use and PLC risk.

Begg’s test and Egger’s test were used to test publication bias when there were 10 or more included studies. We conducted all statistical analyses using STATA software (version 12.0, StataCorp, College Station, TX). Statistical significance level was set at *P* < 0.05 under two-sided test unless otherwise specified.

## Results

### Literature search

**Figure 1:**
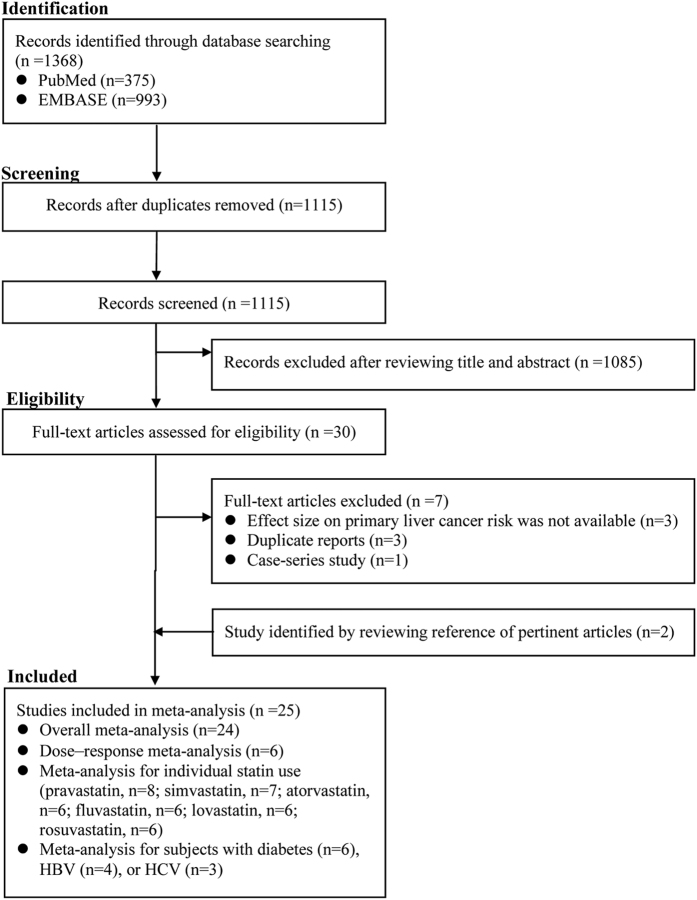
The flowchart of identifying relevant studies.

Our electronic search initially identified 375 citations and 993 citations from PubMed and EMBASE, respectively. A total of 30 citations were thought to be potentially relevant after reviewing titles and abstracts. Seven citations were further excluded after reading carefully the full text. Two studies^32^,^33^ were found to be eligible for inclusion in the process of handsearch. Two studies^16^,^34^ originating from the same cohort were included, because the data they provided could be used to do different analyses (one for overall meta-analysis^34^, the other for dose–response analysis^16^). Thus, 25 studies were included in this meta-analysis (Fig. 1).

### Study characteristics

The results for study characteristics are shown in Supplementary Tables S1–S4. Twelve studies were conducted in Asia^13^,^14^,^15^,^18^,^19^,^20^,^21^,^22^,^23^,^26^,^35^,^36^, and remaining studies were conducted in western countries^12^,^16^,^17^,^29^,^32^,^33^,^34^,^37^,^38^,^39^,^40^,^41^,^42^. Among included studies, 12 were cohort studies^13^,^14^,^15^,^16^,^26^,^29^,^33^,^34^,^35^,^36^,^38^,^42^, 6 were case-control studies^18^,^19^,^20^,^32^,^37^,^41^, 4 were nested case-control studies^12^,^17^,^21^,^40^ and remaining 3 were post hoc analyses of RCTs^22^,^23^,^39^ of statin use and cardiovascular diseases. Our study involved 2,176,213 individuals, consisting of 1,330,795 men (61.2%) and 845,418 women (38.8%). Eighteen studies recruited subjects from population registers and remaining 7 were hospital-based^21^,^22^,^23^,^26^,^32^,^39^,^40^. Most studies were conducted in the general population, whereas 4 studies^13^,^15^,^26^,^35^ were conducted in population with HBV infection, 4^ ^14,16,34,41 in those with HCV infection, and 2^ ^21,40 in those with diabetes. Most studies defined non-user as subjects with no statin prescription, whereas 10 studies^12^,^13^,^14^,^15^,^16^,^34^,^35^,^36^,^37^,^42^ defined non-users as subjects with low cumulative dose of statins during study period. Adjusted risk estimates were available for all studies except one post hoc analysis of RCTs^39^ and one case-control study^32^. As for quality assessment, 18 studies were found to be of high quality, indicating the quality of included studies was generally good.

### Overall meta-analysis

**Figure 2:**
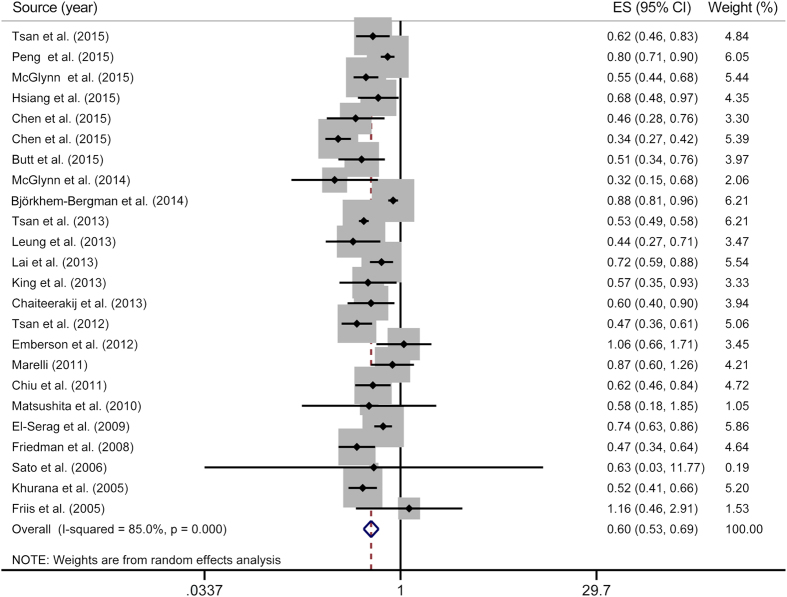
Overall meta-analysis on statin use and primary liver cancer risk. The squares represent the risk estimate for each individual study, with the area reflecting the weight assigned to the study. The horizontal line across each square represents the 95% confidence interval. The diamond represents the summary risk estimate, with width representing 95% confidence interval. CI, confidence interval; ES, effect size.

On the basis of 24 studies, compared with statin non-users, statin users experienced a significantly decreased risk for developing PLC (RR = 0.60, 95% CI = 0.53**–**0.69), with substantial heterogeneity (*P*_heterogeneity_ < 0.01, *I*^*2*^ = 85.0%) (Fig. 2).

### Dose–response meta-analyses

**Figure 3:**
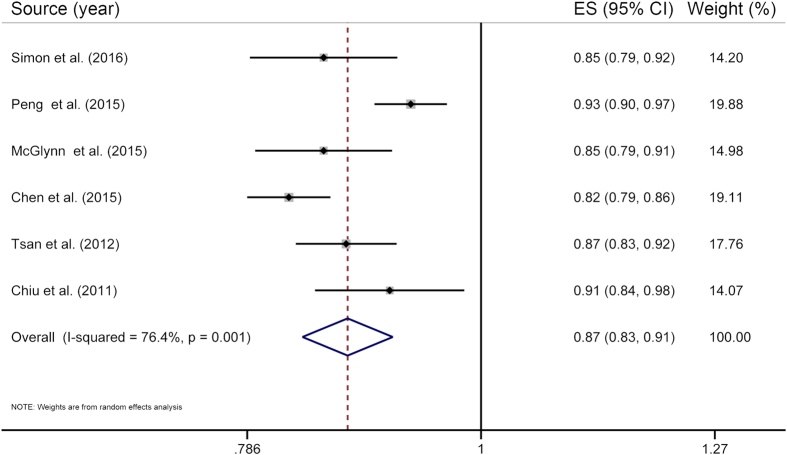
The two-stage dose–response meta-analysis on statin use and the primary liver cancer risk. The squares represent the risk estimate for each individual study, with the area reflecting the weight assigned to the study. The horizontal line across each square represents the 95% confidence interval. The diamond represents the summary risk estimate, with width representing 95% confidence interval. CI, confidence interval; ES, effect size.

**Figure 4:**
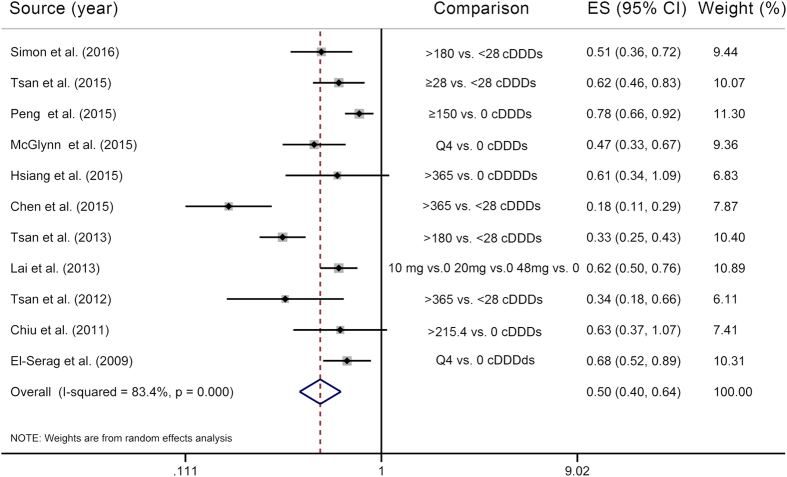
Highest versus lowest meta-analysis on statin use and the primary liver cancer risk. The squares represent the risk estimate for each individual study, with the area reflecting the weight assigned to the study. The horizontal line across each square represents the 95% confidence interval. The diamond represents the summary risk estimate, with width representing 95% confidence interval. CI, confidence interval; cDDDs, cumulative defined daily doses; ES, effect size.

**Figure 5:**
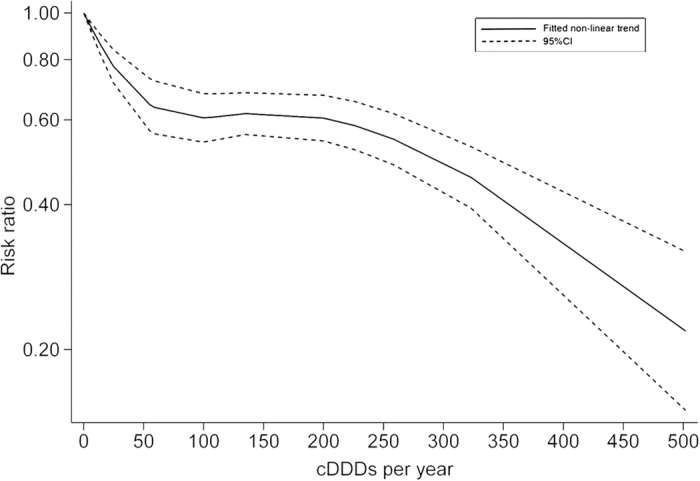
Non-linear dose–response analysis on statin use and the primary liver cancer risk. CI, confidence interval; cDDDs, cumulative defined daily doses.

Six studies^12^,^13^,^15^,^16^,^19^,^20^ were included in the two-stage dose–response meta-analysis on statin use and PLC risk, involving 8,530 PLC cases and 118,961 subjects, and corresponding results showed that for every 50 cDDDs per year increment in statin dose, the PLC risk significantly decreased by 13% (RR = 0.87, 95% CI = 0.83**–**0.91), with substantial heterogeneity (*P*_heterogeneity_ < 0.01, *I*^*2*^ = 76.4%) (Fig. 3). Highest versus lowest meta-analysis included a total of 11 studies^12^,^13^,^14^,^15^,^16^,^18^,^19^,^20^,^26^,^35^,^40^, involving 45,335 PLC cases and 548,518 subjects, and found that the combined RR for PLC was 0.50 (95% CI = 0.40**–**0.64), with substantial heterogeneity (*P*_heterogeneity_ < 0.01, *I*^*2*^ = 83.4%) (Fig. 4). Using restricted cubic spline function, we found a potential non-linear dose–response association between statin use and PLC risk (*P*_non-linearity_ < 0.01) (Fig. 5). The non-linear curve showed that there was a dose–response association between statin dose and decreased risk of PLC below approximately 100 cDDDs per year or above approximately 200 cDDDs per year, whereas the PLC risk did not decrease further between 100 and 200 cDDDs per year. Compared with statin non-use, the estimated RRs for PLC were 0.77 (95% CI = 0.71**–**0.84) for 25 cDDDs per year, 0.65 (95% CI = 0.57**–**0.73) for 55 cDDDs per year, 0.60 (95% CI = 0.54**–**0.67) for 200 cDDDs per year, 0.46 (95% CI = 0.39**–**0.53) for 320 cDDDs per year, and 0.22 (95% CI = 0.15**–**0.32) for 500 cDDDs per year.

### Meta-analyses for individual statins

The results of meta-analyses for individual statins are summarized in Supplementary Table S5. All statins studied were found to be significantly associated with a reduced risk of PLC. Specifically, the pooled RRs for PLC were 0.71 (95% CI = 0.58**–**0.88) for pravastatin, 0.61 (95% CI = 0.54**–**0.69) for simvastatin, 0.53 (95% CI = 0.42**–**0.67) for atorvastatin, 0.65 (95% CI = 0.52**–**0.81) for fluvastatin, 0.49 (95% CI = 0.34**–**0.69) for rosuvastatin, and 0.65 (95% CI = 0.56**–**0.74) for lovastatin.

### Meta-analyses for individuals with diabetes, HBV or HCV

**Figure 6:**
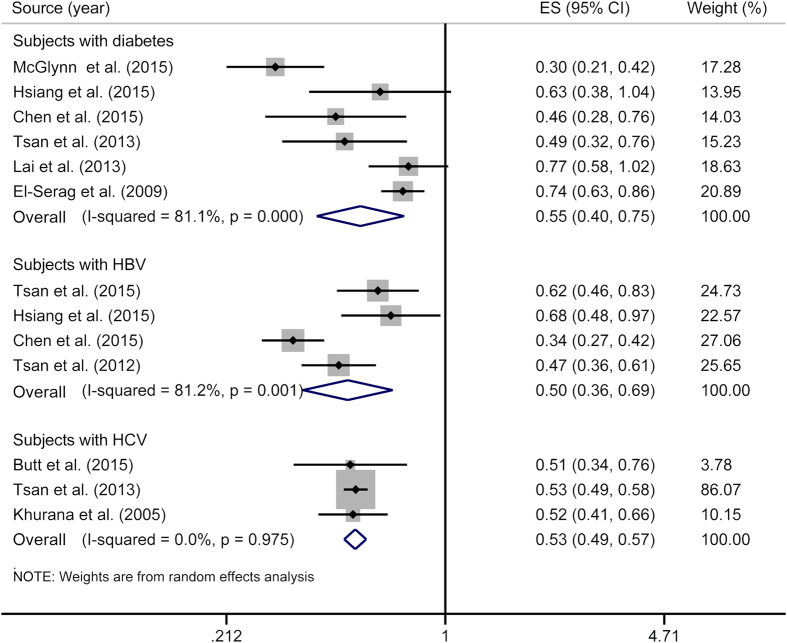
Meta-analyses for individuals with diabetes, HBV or HCV. The squares represent the risk estimate for each individual study, with the area reflecting the weight assigned to the study. The horizontal line across each square represents the 95% confidence interval. The diamond represents the summary risk estimate, with width representing 95% confidence interval. HBV, hepatitis B virus; HCV, hepatitis C virus. CI, confidence interval; ES, effect size.

The overall RRs for PLC were 0.55 (95% CI = 0.40**–**0.75; *P*_heterogeneity_ < 0.01, *I*^*2*^ = 81.1%) for individuals with diabetes, 0.50 (95% CI = 0.36**–**0.69; *P*_heterogeneity_ < 0.01, *I*^*2*^ = 81.2%) for those with HBV infection, and 0.53 (95% CI = 0.49**–**0.57; *P*_heterogeneity_ = 0.98, *I*^*2*^ = 0.0%) for those with HCV infection (Fig. 6).

### Subgroup analyses

The results of subgroup analyses are summarized in Supplementary Table S6. Overall, the significantly inverse association between statin use and PLC risk remained in all subgroups, except in post hoc analyses of RCTs, or in studies with unadjusted risk estimates. A significant difference between subgroups stratified by risk level of developing PLC was detected (*P*_interaction_ = 0.02).

### Sensitivity analyses

The results of sensitivity analyses are present in Supplementary Table S7 and Supplementary Fig. S1. Ignoring a single study in turn did not significantly alter the initial relationship of statin use with PLC risk, with the pooled RR ranging from 0.59 (95% CI = 0.52**–**0.66)^37^ to 0.62 (95% CI = 0.55**–**0.70)^13^. Repeating meta-analysis through a fixed-effects model and using crude risk estimates produced a RR of 0.65 (95% CI = 0.63**–**0.68) and 0.61 (95% CI = 0.52**–**0.72), respectively. Restricting meta-analysis to studies defining statin non-user as subjects with no statin prescription yielded a similar risk estimate (RR = 0.68, 95% CI = 0.60**–**0.76), with the *I*^*2*^ statistic dropping from 85.0% to 50.1%.

### Publication bias

There was no evidence of publication bias for any association as revealed by Begg’s test and Egger’s test (all *P* > 0.05).

## Discussion

Findings from the overall meta-analysis supported an inverse association between statin use and PLC risk, which are consistent with previous meta-analyses^11^,^24^,^25^. Our two-stage dose–response analysis indicated that higher statin dose was associated with a lower risk of PLC. We also found evidence of a potential non-linear dose–response association between statin use and PLC risk. Our subgroup analyses revealed more significant reduction in PLC risk by statins in high- versus non-high-risk populations (high-risk population refers to subjects with HBV infection or HCV infection). Interestingly, as shown by meta-analyses for individual statins, the protective effect of rosuvastatin on PLC risk was more pronounced compared with other studied statins. In addition, we found that the inverse association between statin use and PLC risk persisted in patients with diabetes, HBV infection, or HCV infection.

The statin class is comprised of some heterogeneous medications varying in properties. Considering this fact, individual statins may have different chemopreventive effects. In the present study, we found that all studied statins reduced the risk of PLC, and the risk reduction was more evident with rosuvastatin, a hydrophilic statin. The latter finding is of particular interest, and is inconsistent with the perspective that the chemopreventive effect of lipophilic statins against cancer may be greater than that of hydrophilic statins^43^. The underlying mechanisms for the pronounced risk reduction by rosuvastatin may be related to its unique chemical properties, which make it have the highest affinity for 3-hydroxy-3-methylglutaryl coenzyme A reductase among commonly used statins^44^. In fact, in addition to the chemopreventive effect observed in our study, several studies have consistently suggested that rosuvastatin presents greater efficacy in reducing low-density lipoprotein cholesterol concentrations compared with all other available statins^45^,^46^,^47^. Rosuvastatin is the most prescribed brand name drug in the USA currently, and findings from the present study possibly encourage physicians continuing to prescribe this drug.

Our comprehensive search did not identify any RCTs of statins where the incidence of PLC was a primary outcome. In fact, considering huge costs and ethical concerns, conducting such a study is almost impossible. Nevertheless, the present meta-analysis identified 3 post hoc analyses of RCTs of statin use and cardiovascular diseases^22^,^23^,^39^. We observed that pooled risk estimates on statin use and PLC risk reached statistical significance for observational studies, but not for post hoc analyses of RCTs, which was not unexpected given weaknesses of post hoc analyses of RCTs. A major weakness is the insufficient follow-up duration, with the longest follow-up duration of only 5.1 years among these 3 studies^22^. Consequently, they observed few incident PLC cases, and thus had limited power to examine any effects of statin use on PLC risk. Indeed, only a total of 81 PLC cases were observed during their entire study period. Generally, for many types of cancer, the ideal follow-up duration of a trial in relation to cancer prevention should be equal to or more than 10 years, which can produce an ample number of cancer cases^48^. When it comes to PLC, however, it may be longer considering the low incidence of PLC in the general population. Another weakness is that the incidence of PLC was a secondary outcome. As a result, data on statin use and PLC risk from these 3 post hoc analyses of RCTs were not systematically collected, and thus might be driven by ascertainment bias. The difference of risk estimates between observational studies and post hoc analyses of RCTs has also been observed in other studies^10^,^49^.

In the present study, substantial heterogeneity was observed in the overall meta-analysis. Of included studies, definitions of statin non-users presented high diversity, which might explain the observed heterogeneity to some extent. Indeed, as shown by our sensitivity analysis, moderate heterogeneity was observed when restricting our analysis to studies where statin non-users referred to individuals with no statin prescription. In addition, our subgroup analysis suggested that the risk level of developing PLC was another possible source of heterogeneity. Specifically, there was more significant reduction in PLC risk by statins in high- versus non-high-risk populations. The precise mechanism behind this phenomenon remains unknown, which possibly involves interactions between statins and hepatitis viruses. Indeed, *in vitro* studies have found that statins are capable of inhibiting HBV and HCV replication^50^,^51^. The exact mechanism of the antiviral effect of statins is still unclear, which may be associated with the inhibition of geranylgeranylation of cellular proteins^52^. On the basis of the above benefits as well as the proven efficacy and safety of statins in patients with chronic liver disease^53^, healthcare practitioners should discontinue withholding statins from those with chronic liver disease.

The protective effect of statins on PLC risk involves several potential mechanisms. It is well-known that statins can competitively inhibit the activity of 3-hydroxy-3-methylglutaryl coenzyme A reductase, a rate-limiting enzyme in the mevalonate synthesis pathway. Consequently, the synthesis of not only cholesterol but also isoprenoids is inhibited. The isoprenoids, mainly including farnesyl pyrophosphate and geranylgeranyl pyrophosphate, are required for prenylation of Ras and Rho oncoproteins^54^ that play a pivotal role in cell proliferation and survival^55^. Thus, the inactivation of Ras and Rho oncoproteins resulting from the inhibited synthesis of isoprenoids possibly contributes to cell apoptosis. Of note, it is also suggested that statins may exert their proapoptotic effects through altering the levels of Bcl-2 family members^56^. Statins can modulate both activity and expression of cell cycle regulating proteins, including cyclins, as well as cyclin-dependent kinases and their inhibitors, finally resulting in inhibition of cell proliferation^54^. In addition to the aforementioned mechanisms, in hepatocellular cancer cell lines, it is observed that atorvastatin can block both Myc (an oncogene closely related to hepatocarcinogenesis) phosphorylation and activation, and thus suppress tumor initiation and growth^57^.

Several limitations of this study should be acknowledged. First, the common side effects of statins include hepatotoxicity, myopathy, myoglobinuria as well as acute renal failure^58^. Many healthcare workers avoid prescribing statins to patients with chronic liver disease for fear of the occurrence of potential hepatotoxicity. This fact raises a concern that the observed inverse association of statin use with PLC risk might be explained by the confounding by indication. Nonetheless, some individual studies have found that statins reduce the risk of PLC in patients with and without chronic liver disease^12^,^18^, which possibly attenuates the concern to some degree. In addition, considering most included cohort studies employed a time-fixed analysis to quantify the association of statin use with PLC risk, the observed protective effect of statins on PLC risk might attribute to immortal time bias^59^. Nevertheless, Hsiang and colleagues^26^ observed that statin use was still associated with a reduced risk of hepatocellular carcinoma after eliminating this bias with the landmark analysis method, indicating that immortal time bias did not play a major role in the association between statin use and PLC risk. Second, many included studies attempted to adjust various potential confounders, but the possibility of residual confounding cannot be ruled out, considering that our findings are derived predominantly from observational studies where residual confounding always exists. Third, in the present study, publication bias was tested with formal statistical tests only in the case of ≥10 included studies. Therefore, we cannot exclude the possibility that the results from meta-analyses involving <10 studies are driven by publication bias. Finally, we observed substantial heterogeneity for the present meta-analysis. Such heterogeneity raises some concerns on the reliability of our pooled results. Nevertheless, clinical and methodological heterogeneity exist for all meta-analyses, especially meta-analysis of observational studies. Moreover, we have identified the sources of the observed heterogeneity through sensitivity and subgroup analyses.

In summary, our study suggests a protective effect of statins on PLC risk, most notably of rosuvastatin. This protective effect is more pronounced in high-risk populations, presents a dose–dependent manner, and remains in patients with diabetes, HBV infection, or HCV infection. These findings appear to support that satins can serve as chemopreventive agents of PLC to reduce the public health burden of this malignancy. However, whether the observed inverse association of statin use with PLC risk is causal or is spurious due to potential biases remains uncertain. Prospective cohort studies with sufficient follow-up duration and large sample size are therefore warranted to address this issue before formally recommending statins for preventing PLC.

## Additional Information

**How to cite this article**: Zhong, G.-C. *et al*. Meta-analysis of studies using statins as a reducer for primary liver cancer risk. *Sci. Rep.* **6**, 26256; doi: 10.1038/srep26256 (2016).

## Supplementary Material

Supplementary Information
