# Supplementary material for: Meta-analysis of studies using statins as a reducer for primary liver cancer risk
Source: Sci Rep. 2016 May 20;6:26256. doi: 10.1038/srep26256 (PMC4873806; doi:10.1038/srep26256)
Supplement: Supplementary Information [file srep26256-s1.pdf]

**Meta-analysis of studies using statins as a reducer for primary liver cancer risk**

Guo-Chao Zhong, Yan Liu, Yuan-Yuan Ye, Fa-Bao Hao, Kang Wang & Jian-Ping Gong

**Supplementary List 1 Search Strategy**

**PubMed**

**#1** (((cancer*[Title/Abstract]) OR neoplasm*[Title/Abstract]) OR carcinoma*[Title/Abstract]) OR malignanc*[Title/Abstract]

**#2** ((liver[Title/Abstract]) OR hepatic[Title/Abstract]) OR hepatocellular[Title/Abstract]

**#3** **#1** AND **#2**

**#4** ((hepatocarcinoma[Title/Abstract]) OR "liver cell carcinoma*"[Title/Abstract]) OR hepatoma[Title/Abstract]

**#5** **#3** OR **#4**

**#6** (((((((((statin*[Title/Abstract]) OR "HMG-CoA reductase inhibitor*"[Title/Abstract]) OR "Hydroxymethylglutaryl-CoA reductase inhibitor*"[Title/Abstract]) OR atorvastatin[Title/Abstract]) OR cerivastatin[Title/Abstract]) OR fluvastatin[Title/Abstract]) OR lovastatin[Title/Abstract]) OR pravastatin[Title/Abstract]) OR rosuvastatin[Title/Abstract]) OR simvastatin[Title/Abstract]) OR

pitavastatin[Title/Abstract]

**#7** **#5** AND **#6**

**EMBASE**

**#1** statin* AND ([article]/lim OR [article in press]/lim OR [conference abstract]/lim OR [conference paper]/lim) AND ([chinese]/lim OR [english]/lim) AND [embase]/lim

**#2** 'hmg coa' AND reductase AND inhibitor* AND ([article]/lim OR [article in press]/lim OR [conference abstract]/lim OR [conference paper]/lim) AND ([chinese]/lim OR [english]/lim) AND [embase]/lim

**#3** 'hydroxymethylglutaryl coa' AND reductase AND inhibitor* AND ([article]/lim OR [article in press]/lim OR [conference abstract]/lim OR [conference paper]/lim) AND ([chinese]/lim OR [english]/lim) AND [embase]/lim

**#4** atorvastatin AND ([article]/lim OR [article in press]/lim OR [conference abstract]/lim OR [conference paper]/lim) AND ([chinese]/lim OR [english]/lim) AND [embase]/lim

**#5** cerivastatin AND ([article]/lim OR [article in press]/lim OR [conference abstract]/lim OR [conference paper]/lim) AND ([chinese]/lim OR [english]/lim) AND [embase]/lim

**#6** fluvastatin AND ([article]/lim OR [article in press]/lim OR [conference abstract]/lim OR [conference paper]/lim) AND ([chinese]/lim OR [english]/lim) AND [embase]/lim

**#7** lovastatin AND ([article]/lim OR [article in press]/lim OR [conference abstract]/lim OR [conference paper]/lim) AND ([chinese]/lim OR [english]/lim) AND [embase]/lim

**#8** pravastatin AND ([article]/lim OR [article in press]/lim OR [conference abstract]/lim OR [conference paper]/lim) AND ([chinese]/lim OR [english]/lim) AND [embase]/lim

**#9** rosuvastatin AND ([article]/lim OR [article in press]/lim OR [conference abstract]/lim OR [conference paper]/lim) AND ([chinese]/lim OR [english]/lim) AND [embase]/lim

**#10** simvastatin AND ([article]/lim OR [article in press]/lim OR [conference abstract]/lim OR [conference paper]/lim) AND ([chinese]/lim OR [english]/lim) AND [embase]/lim

**#11** pitavastatin AND ([article]/lim OR [article in press]/lim OR [conference abstract]/lim OR [conference paper]/lim) AND ([chinese]/lim OR [english]/lim) AND [embase]/lim

**#12** **#1** OR **#2** OR **#3** OR **#4** OR **#5** OR **#6** OR **#7** OR **#8** OR **#9** OR **#10** OR **#11**

**#13** cancer* AND ([article]/lim OR [article in press]/lim OR [conference abstract]/lim OR [conference paper]/lim) AND ([chinese]/lim OR [english]/lim) AND [embase]/lim

**#14** neoplasm* AND ([article]/lim OR [article in press]/lim OR [conference abstract]/lim OR [conference paper]/lim) AND ([chinese]/lim OR [english]/lim) AND [embase]/lim

**#15** carcinoma* AND ([article]/lim OR [article in press]/lim OR [conference abstract]/lim OR [conference paper]/lim) AND ([chinese]/lim OR [english]/lim) AND [embase]/lim

**#16** malignanc* AND ([article]/lim OR [article in press]/lim OR [conference abstract]/lim OR [conference paper]/lim) AND ([chinese]/lim OR [english]/lim) AND [embase]/lim

**#17** **#13** OR **#14** OR **#1**5 OR **#16**

**#18** liver AND ([article]/lim OR [article in press]/lim OR [conference abstract]/lim OR [conference paper]/lim) AND ([chinese]/lim OR [english]/lim) AND [embase]/lim

**#19** hepatic AND ([article]/lim OR [article in press]/lim OR [conference abstract]/lim OR [conference paper]/lim) AND ([chinese]/lim OR [english]/lim) AND [embase]/lim

**#20** hepatocellular AND ([article]/lim OR [article in press]/lim OR [conference abstract]/lim OR [conference paper]/lim) AND ([chinese]/lim OR [english]/lim) AND [embase]/lim

**#21** **#18** OR **#19** OR **#20**

**#22** **#17** AND **#21**

**#23** hepatocarcinoma AND ([article]/lim OR [article in press]/lim OR [conference abstract]/lim OR [conference paper]/lim) AND ([chinese]/lim OR [english]/lim) AND [embase]/lim

**#24** liver AND cell AND carcinoma* AND ([article]/lim OR [article in press]/lim OR [conference abstract]/lim OR [conference paper]/lim) AND ([chinese]/lim OR [english]/lim) AND [embase]/lim

**#25** hepatoma AND ([article]/lim OR [article in press]/lim OR [conference abstract]/lim OR [conference paper]/lim) AND ([chinese]/lim OR [english]/lim) AND [embase]/lim

**#26** **#23** OR **#24** OR **#25**

**#27** **#22** OR **#26**

**#28** **#12** AND **#27**

**Supplementary Table S1.** Characteristics of included cohort studies, nested case-control studies, and post hoc analyses of RCTs on statin use and primary liver cancer risk

| Study; location | Design | Age (y) [a] | Sample size | Data source | Study population | Definition of nonuser | Cancer cases | Follow-up [a] (y) | Adjustment factors |
|---|---|---|---|---|---|---|---|---|---|
| Simon et al., 2016; USA [16] | Cohort | 53.0 | T: 9135<br>M:8745<br>W:390 | Population-based | Subjects with HCV | Subjects using statins ≤28 cDDDs | 239 | 7.4 | Age, sex, alcohol, ACEIs, attainment of sustained virologic response, baseline FIB-4 score, BMI, caffeine intake, daily caffeine intake , diabetes, metformin use, NSAIDs, other lipid-lowering agent use, prior completed HCV treatment, race, smoking |
| Tsan et al., 2015; Taiwan [35] | Cohort | 35.0 [b] | T:91265<br>M:66635<br>W:24630 | Population-based | Subjects with HBV | Subjects using statins <28 cDDDs | 2841 | 2.9 | Interferon, income, urbanization |
| McGlynn et al., 2015; UK [12] | Nested case-control | 67.0 | T:5835<br>M:4178<br>W:1657 | Population-based | General population | Subjects with one or no statin prescription | 1195 | ≤23.0 | Alcohol-related disorders, BMI, diabetes, HBV, HCV, smoking, metabolic disorders, use of antidiabetics, use of |

| | | | | | | | | | |
|---|---|---|---|---|---|---|---|---|---|
| | | | | | | | | | aspirin, use of paracetamol |
| Hsiang et al., 2015; China [26] | Cohort | 38.1 | T:53513<br>M:14145<br>W:39368 | Hospital-based | Subjects with HBV | Subjects with no statin prescription | 1298 | 4.6 [b] | Age, sex, albumin, ALT, comorbidities, creatinine, medications, platelet count, total bilirubin |
| Chen et al., 2015; Taiwan [21] | Nested case-control | 62.6 | T:1700<br>M:1325<br>W:375 | Hospital-based | Subjects with diabetes | Unclear | 340 | ≤11.0 | Alcoholic liver damage, HCV, HBV, cirrhosis duration |
| Chen et al., 2015; Taiwan [13] | Cohort | NA | T:61898<br>M:35595<br>W:26303 | Population-based | Subjects with HBV | Subjects using statins <28 cDDDs | 1735 | ≤9.0 | Age, sex, acetylcholinesterase inhibitors, anti-HBV drugs, area, aspirin, comorbidities, index year, nonstatin lipid-lowering drugs |
| Butt et al., 2015; USA [34] | Cohort | 53.0 for statin users; 52.0 for statin non-users [b] | T:7248<br>M:6929<br>W:319 | Population-based | Subjects with HCV | Subjects using statins ≤14 days or with no statin prescription | NA | ≥2.0 | FIB-4 score |

| | | | | | | | | | |
|---|---|---|---|---|---|---|---|---|---|
| McGlynn et al., 2014; USA [17] | Nested case-control | NA | T:562 M:418 W:144 | Population -based | General population | Subjects with no statin prescription | 94 | 8.1 | Alcohol-related disorders, chronic obstructive pulmonary disease, diabetes, HCV, hypertension, race |
| Tsan et al., 2013; Taiwan [14] | Cohort | 50.4 [b] | T:260864 M:128263 W:132601 | Population -based | Subjects with HCV | Subjects using statins <28 cDDDs | 27883 | 10.7 | Age, sex, diabetes, income, liver cirrhosis, urbanization |
| Leung et al., 2013; Taiwan [36] | Cohort | 58.3 | T:34205 M:16150 W:18055 | Population -based | General population | Subjects with one or no statin prescription | 424 | 4.2 | Age, sex, cardiovascular drugs, Charlson score, comorbidities, hormone-replacement therapy, non-statin lipid-lowering drugs, NSAIDs |
| King et al., 2013; USA [38] | Cohort | NA | T:136178 M:39634 W:96544 | Population -based | General population | Unclear | 125 | ≤20.0 | Age, alcohol, aspirin, BMI, diabetes, smoking |
| Tsan et al., 2012; Taiwan [15] | Cohort | 35.6 [b] | T:33413 M:19442 W:13971 | Population -based | Subjects with HBV | Subjects using statins <28 cDDDs | 1021 | 9.8 | Age, sex, diabetes, income, liver cirrhosis, urbanization |
| Emberson et al., 2012; Europe, Australia, USA [39] | Post hoc analysis of RCTs | 63.0 | T:134537 M:95529 W:39008 | Hospital-based | General population | Subjects with no statin prescription | 68 | 4.8 | None |
| Marelli et al., 2012; USA [33] | Cohort | 64.2 | T:91714 M:48059 | Population -based | General population | Subjects with no statin | 105 | 4.7 | Age, sex, comorbidities, BMI, race, smoking |

| | | | | | | | | | |
|---|---|---|---|---|---|---|---|---|---|
| | | | W:43655 | | | prescription | | | |
| Matsushita et al., 2010; Japan [22] | Post hoc analysis of RCTs | 57.6 | T:13724<br>M:7082<br>W:6642 | Hospital-based | General population | Subjects with no statin prescription | 12 | 5.1 | Age, sex, smoking |
| El-Serag et al., 2009; USA [40] | Nested case-control | 72.0 | T:6515<br>M:6430<br>W:85 | Hospital-based | Subjects with diabetes | Subjects with no statin prescription | 1303 | 2.4 | ACEIs, alcoholic liver disease, alcoholism, cirrhosis, HBV, HCV, anti-HCV drugs, NSAIDs, propensity to use statins, race |
| Friedman et al., 2008; USA [29] | Cohort | NA | T:361859<br>M:192598<br>W:169261 | Population-based | General population | Subjects with no statin prescription | 42 | 4.9 [b] | Calendar year |
| Sato et al.,2006; Japan [23] | Post hoc analysis of RCTs | NA | T:263<br>M:215<br>W:48 | Hospital-based | Subjects with CHD | Subjects using statins for ≤25% of study period | 1 | 4.8 | Age, sex, pravastatin medicated, smoking, total serum cholesterol |
| Friis et al., 2005; Denmark [42] | Cohort | 47.1 | T:348262<br>M:175775<br>W:172487 | Population-based | General population | Subjects with one or no statin prescription | 171 | 3.5 | Age, sex, calendar period, cardiovascular drugs, hormone-replace treatment, NSAIDs |

Abbreviations: ACEIs, angiotensin converting enzyme inhibitor; ALT, alanine aminotransferase; BMI, body mass index; cDDDs, cumulative defined daily doses; CHD, coronary heart disease; HBV, hepatitis B virus; HCV, hepatitis C virus; M: men; NA, not available; NSAIDs, nonsteroidal anti-inflammatory drugs; RCT, randomized controlled trial; T, total; W: women.

[a] Mean value unless otherwise specified.

[b] Median value.

**Supplementary Table S2.** Characteristics of included case-control studies on statin use and primary liver cancer risk

| Study; location | Study period | Age (y) [a] | Sample size | Data source | Study population | Definition of nonuser | Cases/ Controls | Adjustment factors |
|---|---|---|---|---|---|---|---|---|
| Peng et al., 2015; Taiwan [20] | 2002-2011 | 68.0 | T:6348<br>M:3241<br>W:3107 | Population-based | General population | Subjects with no statin prescription | 3174/ 3174 | Age, sex, aspirin, alcohol-related illness, biliary tract disease, coronary artery disease, Charlson comorbidity index score, chronic pancreatitis, cirrhosis, chronic obstructive pulmonary disease, diabetes, gastric disease, haemochromatosis, HBV, HCV, inflammatory bowel disease, stroke, metformin |
| Björkhem-Bergman et al., 2014; Sweden [37] | 2006-2010 | NA | T:23964<br>M:12456<br>W:11508 | Population-based | General population | Subjects using statins ≤9 months or with no statin prescription | 3994/ 19970 | Age, sex, acetylsalicylic acid, alcohol hepatitis, chemotherapy, diabetes, education, liver disease, NSAIDs, treatment with cortisone, viral hepatitis |
| Lai et al., 2013; Taiwan [18] | 2000-2009 | 62.6 | T:17400<br>M:12625<br>W:4775 | Population-based | General population | Subjects with no statin prescription | 3480/ 13920 | Age, sex, alcoholic liver damage, cirrhosis, diabetes, duration of statin use, HBV, HCV, non-alcoholic fatty liver disease, non-statin lipid-lowering drug, drugs for HBV and HCV, use of metformin, use of thiazolidinedione |

| | | | | | | | | |
|---|---|---|---|---|---|---|---|---|
| Chaiteerakij et al., 2013; USA [32] | 2000-2010 | 61.4 | T:421<br>M:208<br>W:213 | Hospital-based | General population | Subjects with no statin prescription | 165/<br>256 | None |
| Chiu et al., 2011; Taiwan [19] | 2005-2008 | 66.0 | T:2332<br>M1606<br>W:726 | Population-based | General population | Subjects with no statin prescription | 1166/<br>1166 | Sex, alcoholic liver disease, birth year, cirrhosis, diabetes, HBV, HCV, index date, number of hospitalizations, use of non-statin lipid-lowering drugs |
| Khurana et al., 2005;USA [41] | 1998-2004 | 61.1 | T:480306<br>M:440441<br>W:39865 | Population-based | Subjects with HCV | NA | 409/<br>479897 | Age, HCV |

Abbreviations: cDDDs, cumulative defined daily doses; HBV, hepatitis B virus; HCV, hepatitis C virus; M: men; NA, not available; NSAIDs, nonsteroidal anti-inflammatory drugs; T, total; W: women.

[a] Mean value.

**Supplementary Table S3.** The Results of quality assessment for cohort studies and post hoc analyses of RCTs

| Study | Selection | | | | Comparability | Outcome | | | Total Score |
|---|---|---|---|---|---|---|---|---|---|
| | Representativeness of exposed cohort ☆ | Selection of non-exposed cohort ☆ | Exposure ascertainment ☆ | No history of disease ☆ | Comparable on confounders ☆☆ | Outcome Assessment ☆ | Adequate follow-up (≥10y) ☆ | Loss to follow-up rate (≤20%) ☆ | |
| Simon et al. 2016 [16] | ☆ | ☆ | ☆ | ☆ | ☆ ☆ | ☆ | | ☆ | 8 |
| Tsan et al. 2015 [35] | ☆ | ☆ | | ☆ | ☆ | | | ☆ | 5 |
| Hsiang et al. 2015 [26] | ☆ | ☆ | ☆ | ☆ | ☆ ☆ | | | ☆ | 7 |
| Chen et al. 2015 [13] | ☆ | ☆ | ☆ | ☆ | ☆ ☆ | | | ☆ | 7 |
| Butt et al. 2015 [34] | ☆ | ☆ | ☆ | ☆ | ☆ | ☆ | | ☆ | 7 |
| Tsan et al. 2013 [14] | ☆ | ☆ | ☆ | ☆ | ☆ | ☆ | ☆ | ☆ | 8 |
| Leung et al. 2013 [36] | ☆ | ☆ | ☆ | ☆ | ☆ | | | ☆ | 6 |
| King et al. 2013 [38] | ☆ | ☆ | | ☆ | ☆ | ☆ | ☆ | ☆ | 7 |
| Tsan et al. 2012 [15] | ☆ | ☆ | ☆ | ☆ | ☆ | ☆ | | ☆ | 7 |
| Emberson et al. 2012 [39] | ☆ | ☆ | | ☆ | ☆ ☆ | ☆ | | ☆ | 7 |
| Marelli et al. 2011 [33] | ☆ | ☆ | ☆ | ☆ | ☆ | ☆ | | ☆ | 7 |
| Matsushita et al. 2010 [22] | ☆ | ☆ | | ☆ | ☆ ☆ | ☆ | | ☆ | 7 |
| Friedman et al. 2008 [29] | ☆ | ☆ | ☆ | ☆ | ☆ | ☆ | | ☆ | 7 |
| Sato et al. 2006 [23] | | ☆ | ☆ | ☆ | ☆ | ☆ | | ☆ | 6 |
| Friis et al. 2005 [42] | ☆ | ☆ | ☆ | ☆ | ☆ | ☆ | | ☆ | 7 |

Abbreviations: RCTs, randomized controlled trials.

**Supplementary Table S4.** The Results of quality assessment for nested case-control studies and case-control study

| Study | Selection | | | | Comparability | Outcome | | | Total Score |
|---|---|---|---|---|---|---|---|---|---|
| | Adequate case definition ☆ | Representativeness of cases ☆ | Selection of controls ☆ | Definition of controls ☆ | Comparable on confounders ☆☆ | Exposure ascertainment ☆ | Ascertainment for cases and controls ☆ | Non-response rate ☆ | |
| Peng et al. 2015 [20] | | ☆ | ☆ | | ☆ ☆ | ☆ | ☆ | | 6 |
| McGlynn et al. 2015 [12] | ☆ | ☆ | ☆ | ☆ | ☆ ☆ | ☆ | ☆ | | 8 |
| Chen et al. 2015 [21] | | ☆ | ☆ | ☆ | ☆ | ☆ | ☆ | | 6 |
| McGlynn et al. 2014 [17] | ☆ | ☆ | ☆ | ☆ | ☆ | ☆ | ☆ | | 7 |
| Björkhem-Bergman et al. 2014 [37] | ☆ | ☆ | ☆ | ☆ | ☆ ☆ | ☆ | ☆ | | 8 |
| Lai et al. 2013 [18] | ☆ | ☆ | ☆ | ☆ | ☆ ☆ | | ☆ | | 7 |
| Chaiteerakij et al. 2013 [32] | ☆ | ☆ | ☆ | ☆ | | ☆ | ☆ | | 6 |
| Chiu et al. 2011 [19] | ☆ | ☆ | ☆ | ☆ | ☆ | ☆ | ☆ | | 7 |
| El-Serag et al. 2009 [40] | ☆ | ☆ | ☆ | ☆ | ☆ ☆ | ☆ | ☆ | | 8 |
| Khurana et al. 2005 [41] | | ☆ | ☆ | ☆ | ☆ | | ☆ | | 5 |

| **Supplementary Table S5.** Individual statin use and primary liver cancer risk | | | | | | | | | |
|---|---|---|---|---|---|---|---|---|---|
| Type of statins | No. of studies | Cancer cases | Sample size | Random-effects model | | Fixed-effects model | | Tests for heterogeneity | |
| | | | | RR | 95% CI | RR | 95% CI | *P* | $I^2$ (%) |
| Pravastatin | 8 | 248 | 15172 | 0.71 | 0.58, 0.88 | 0.70 | 0.59, 0.83 | 0.29 | 17.5 |
| Simvastatin | 7 | 1945 | 10060 | 0.61 | 0.54, 0.69 | 0.62 | 0.57, 0.69 | 0.22 | 27.2 |
| Atorvastatin | 6 | 650 | 4855 | 0.53 | 0.42, 0.67 | 0.61 | 0.55, 0.68 | <0.01 | 70.7 |
| Fluvastatin | 6 | 245 | 1506 | 0.65 | 0.52, 0.81 | 0.69 | 0.58, 0.81 | 0.25 | 24.3 |
| Rosuvastatin | 6 | 202 | 1195 | 0.49 | 0.34, 0.69 | 0.58 | 0.48, 0.70 | 0.10 | 45.8 |
| Lovastatin | 5 | 386 | 2111 | 0.65 | 0.56, 0.74 | 0.65 | 0.56, 0.74 | 0.67 | 0.0 |

Abbreviations: CI, confidence interval; RR, risk ratio.

**Supplementary Table S6.** Subgroup analyses of statin use and primary liver cancer risk based on the overall meta-analysis

| Subgroup | Number of studies | RR | 95% CI | $I^2$ (%) | $P_{heterogeneity}$ | $P_{interaction}$ [a] |
|---|---|---|---|---|---|---|
| All studies | 24 | 0.60 | 0.53, 0.69 | 85.0 | <0.01 | - |
| Study design | | | | | | |
| Cohort study | 11 | 0.53 | 0.46, 0.62 | 66.6 | <0.01 | 0.10 |
| Case-control study | 6 | 0.71 | 0.60, 0.83 | 77.6 | <0.01 | |
| Nested case-control study | 4 | 0.56 | 0.42, 0.75 | 71.0 | 0.02 | |
| Post hoc analysis of RCTs | 3 | 0.96 | 0.62, 1.49 | 0.0 | 0.62 | |
| Source of subjects | | | | | | |
| Population-based | 17 | 0.58 | 0.50, 0.68 | 88.9 | <0.01 | 0.29 |
| Hospital-based | 7 | 0.70 | 0.60, 0.82 | 11.0 | 0.35 | |
| Sex | | | | | | |
| Man | 6 | 0.52 | 0.40, 0.69 | 77.5 | <0.01 | 0.55 |
| Women | 6 | 0.47 | 0.36, 0.60 | 36.0 | 0.17 | |
| Individual age | | | | | | |
| ≥50y | 4 | 0.45 | 0.31, 0.65 | 76.0 | <0.01 | 0.40 |
| < 50y | 4 | 0.53 | 0.42, 0.67 | 0.0 | 0.73 | |
| Sample size | | | | | | |
| ≥10,000 | 15 | 0.61 | 0.50, 0.73 | 89.2 | <0.01 | 0.97 |
| <10,000 | 9 | 0.62 | 0.53, 0.73 | 61.4 | <0.01 | |
| Study location | | | | | | |
| Asian | 12 | 0.56 | 0.47, 0.67 | 83.2 | <0.01 | 0.26 |
| Western | 12 | 0.65 | 0.54, 0.77 | 79.2 | <0.01 | |
| High-risk population [b] | | | | | | |

| | | | | | | |
|---|---|---|---|---|---|---|
| Yes | 7 | 0.51 | 0.43, 0.59 | 67.4 | <0.01 | 0.02 |
| No | 17 | 0.67 | 0.59, 0.76 | 69.3 | <0.01 | |
| Study quality | | | | | | |
| High | 17 | 0.61 | 0.52, 0.72 | 88.0 | <0.01 | 0.78 |
| Low | 7 | 0.59 | 0.47, 0.73 | 66.8 | <0.01 | |
| Adjustment for diabetes | | | | | | |
| Yes | 12 | 0.63 | 0.53, 0.74 | 88.7 | <0.01 | 0.43 |
| No | 12 | 0.57 | 0.46, 0.71 | 75.2 | <0.01 | |
| Adjustment for HCV | | | | | | |
| Yes | 9 | 0.66 | 0.57, 0.77 | 80.6 | <0.01 | 0.24 |
| No | 15 | 0.56 | 0.48, 0.65 | 63.9 | <0.01 | |
| Adjustment for HBV | | | | | | |
| Yes | 8 | 0.63 | 0.51, 0.77 | 91.0 | <0.01 | 0.49 |
| No | 16 | 0.57 | 0.51, 0.64 | 38.0 | 0.06 | |
| Adjustment for confounders | | | | | | |
| Unadjusted | 2 | 0.79 | 0.45, 1.37 | 68.6 | 0.07 | 0.45 |
| 1-3 confounders | 5 | 0.53 | 0.46, 0.62 | 0.0 | 0.78 | |
| 4-6 confounders | 8 | 0.55 | 0.46, 0.67 | 44.1 | 0.08 | |
| ≥7 confounders | 9 | 0.63 | 0.52, 0.77 | 89.9 | <0.01 | |

Abbreviations: CI, confidence interval; HBV, hepatitis B virus; HCV, hepatitis C virus; RCTs, randomized controlled trials; RR, risk ratio.

[a] $P_{\text{interaction}}$ between subgroups was calculated with meta-regression.

[b] “High-risk population” refers to subjects with HBV or HCV infection.

**Supplementary Table S7.** Sensitivity analyses of statin use and primary liver cancer risk

| Category | n | RR | 95% CI | $I^2$ (%) |
|---|---|---|---|---|
| Statistical model | | | | |
| Random-effects model | 24 | 0.60 | 0.53, 0.69 | 85.0 |
| Fixed-effects model | 24 | 0.65 | 0.63, 0.68 | 85.0 |
| Analysis using unadjusted risk estimates | 22 | 0.61 | 0.52, 0.72 | 90.3 |
| Analysis within | | | | |
| Observational studies | 21 | 0.57 | 0.49, 0.67 | 86.5 |
| Studies where non-users refer to persons with no statin prescription | 13 | 0.68 | 0.60, 0.76 | 50.1 |
| Analysis without | | | | |
| Studies with sample size >100,000 | 18 | 0.60 | 0.52, 0.70 | 83.6 |
| Studies with sample size <1,000 | 21 | 0.61 | 0.53, 0.70 | 86.6 |

Abbreviations: CI, confidence interval; RR, risk ratio.

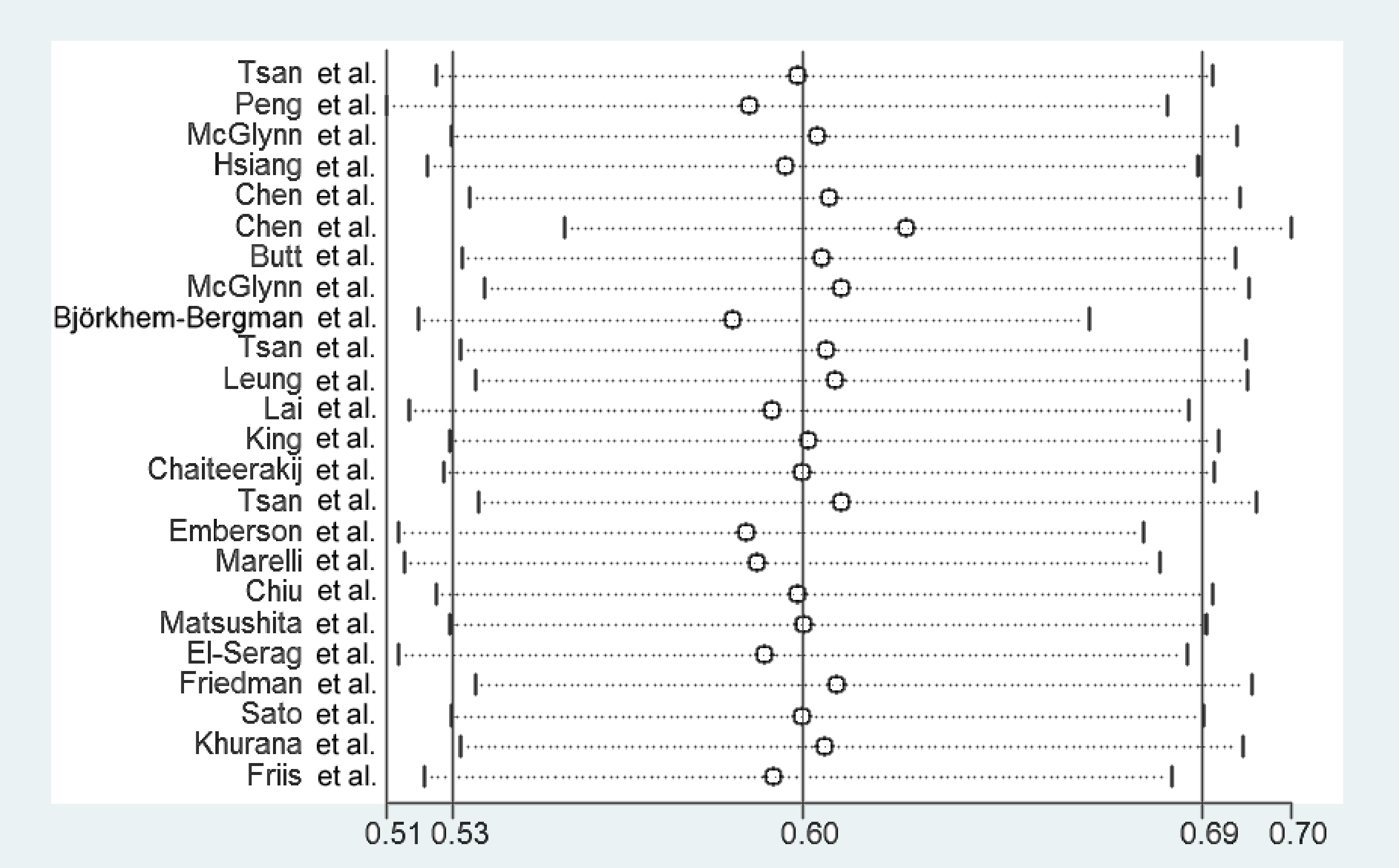


**Supplementary Fig. 1** Sensitivity analysis on parity and primary liver cancer risk: exclusion of a single study in turn. The study cited on the left is the one left out in each turn. The circle represents the summary risk estimates after exclusion of a single study, and the corresponding dot line represents 95% confidence interval. The middle vertical solid line represents summary risk estimates of all included studies, and left and right vertical solid line represent lower limit and upper limit, respectively.
